# Synaptic and Neuronal Autoantibody-Associated Psychiatric Syndromes: Controversies and Hypotheses

**DOI:** 10.3389/fpsyt.2017.00013

**Published:** 2017-02-06

**Authors:** Adam Al-Diwani, Thomas A. Pollak, Alexander E. Langford, Belinda R. Lennox

**Affiliations:** ^1^Department of Psychiatry, University of Oxford, Warneford Hospital, Oxford, UK; ^2^Oxford Health NHS Foundation Trust, Oxford, Oxfordshire, UK; ^3^Department of Psychosis Studies, Institute of Psychiatry, Psychology and Neuroscience, King’s Health Partners, London, UK; ^4^Department of Psychological Medicine, Oxford University Hospitals NHS Foundation Trust, John Radcliffe Hospital, Oxford, UK

**Keywords:** autoimmune diseases of the nervous system, mild encephalitis, glutamatergic neurotransmission, blood–brain barrier disruption, immunotherapy, schizophrenia, bipolar disorder, major depression

## Abstract

Autoimmune encephalitis (AE) mediated by antibodies against synaptic and neuronal surface targets frequently presents with a psychiatric syndrome. In these patients, removal of autoantibodies treats the disease and outcomes are closely linked to early intervention. The discovery of these autoantibodies in isolated psychiatric syndromes has raised the possibility that these patients may derive similar benefits from immunotherapy, a potentially transformational approach to the treatment of mental illness. Although open-label case series suggest impressive therapeutic outcomes, the pathological relevance of these autoantibodies outside of canonical presentations is debated. The advent of diagnostic criteria for AE attempts to facilitate its prompt identification but risks prematurely neglecting the potential scientific and clinical significance of isolated syndromes that do not satisfy these criteria. Here, we propose using a syndrome-level taxonomy that has occasional, but not necessary, overlap with AE: synaptic and neuronal autoantibody-associated psychiatric syndromes or “SNAps”. This will prevent confusion with AE and act heuristically to promote active investigation into this rare example of psychopathology defined on a molecular level. We suggest that this concept would have application in other autoantibody-associated syndromes including seizure, cognitive, and movement disorders, in which similar issues arise. We review putative direct and indirect mechanisms and outline experimentally testable hypotheses that would help to determine prospectively in whom autoantibody detection is relevant, and as important, in whom it is not. We summarize a pragmatic approach to autoantibody testing and management in severe mental illness in order to promptly diagnose AE and advocate a research-orientated experimental medicine paradigm for SNAps, where there is greater equipoise. We conclude that SNAps remains a nascent area of clinical neuroscience with great potential and in ongoing need of psychiatry-led basic and clinical research.

## Introduction

The serum of patients with functional psychoses contains abnormal globulins … I have previously suggested that an autoimmune mechanism might be involved … any interpretation of their meaning is completely speculative and must be approached with the greatest caution.

W. J. Fessel, Autoimmunity and Mental Illness, 1962 (1).

Antibodies that bind to cell surface neuronal, glial, or synaptic targets, collectively known as neural surface antibodies (NSAbs), have attracted significant attention in neurology and psychiatry (2). Their detection in a patient presenting with psychiatric symptoms raises the possibility both of a causal or disease-modifying role and of clinical improvement with immunotherapy (IT). This would represent a major step forward from current largely symptom-targeted psychotropic medications and has been met by clinicians and researchers with enthusiasm.

Although this iteration of autoimmune psychiatry is in its infancy (3), here we argue that there already exists ample evidence to warrant an expanding research program, focusing on robustly establishing the prevalence and relevance of NSAbs in what would otherwise appear to be primary psychiatric disorders. We discuss the controversies in applying knowledge of autoimmune encephalitis (AE) to such psychiatric disorders and suggest experiments by which these controversies may be resolved.

## Are AE and Psychiatric Syndromes Associated with NSAbs Related?

Given the prominence of psychiatric symptoms in many types of AE, and the known importance of receptor targets of NSAbs to psychopathology in psychiatric disorders, there have been extensive efforts to define disease-relevant associations between NSAbs and isolated psychiatric syndromes such as first-episode psychosis (FEP) (4). The development of assays able to detect specific antibodies against central nervous system (CNS) neural surface antigens in combination with careful clinical phenotyping has made this possible. Primary psychiatric disorders, such as schizophrenia, are far more common than AE. If a proportion of these were to have an NSAb-related etiology, then applying the lessons from the rare disease could stand to benefit a far larger group of patients.

Differences in acuity of sample timing, serum testing without paired cerebrospinal fluid (CSF), and variations in assay method have led to a lack of consensus on prevalence of NSAbs in psychiatric illness. Initially, higher rates of autoantibody prevalence were found early in psychotic illnesses compared to the chronic phase (5), but some evidence makes this distinction less clear (6). Live, non-permeabilized cell-based assays (CBAs) largely find higher NSAb prevalence in patients compared to controls (7, 8), but this has not been wholly true of fixed permeabilized CBAs (9–11). The use of CBAs in isolation has been criticized for lacking disease relevance (12); however, some studies supplementing CBA with immunohistochemistry and/or neuronal staining do in fact detect differences between cases and controls in certain psychiatric syndromes such as postpartum psychosis (13).

The rapid development of knowledge on AE, and the increasing and potentially confusing “phenotype spread” associated with NSAbs, have inspired diagnostic criteria for AE (14). These focus on early detection prompting timely IT. Diagnosis is deemed more likely with multiple symptoms, symptoms spanning both neurological and psychiatric domains, and supportive imaging, electroencephalogram (EEG), and CSF changes. Less emphasis is placed on NSAb detection as AEs tend to present stereotypically and can be recognized with readily available investigations, whereas NSAb results may take several weeks to process. However, by definition, psychiatric syndromes with serum NSAb positivity on routinely used CBAs will be incompatible with this system. To illustrate, for NMDAR antibody positivity to be relevant by these criteria, it either needs to be detected in CSF or in serum with multiple assay methods, and “relevance” equates only to a diagnosis of NMDAR-AE. Given that NMDAR antibodies are the most commonly detected NSAb in isolated psychiatric syndromes (9), and that NMDAR-AE occasionally does not progress beyond psychosis, this has implications for much of the field. NSAbs found in isolated psychiatric syndromes, which are currently considered of research interest and potential clinical relevance, would be scientifically neglected or “orphaned.” The reality that CSF is often difficult to obtain in these patients, and multiple assay methods to evaluate serum are rarely available outside specialist centers, compounds this issue.

Furthermore, a negative CSF result should not automatically render NSAb seropositivity irrelevant. First, some NSAbs, such as LGI1, are detected less frequently in CSF, in this case in half or less of LGI1 AE cases (15). Second, if NSAbs are pathogenic in some of the isolated psychiatric presentations in which they are detected, it is likely that they occur at lower titers than in fulminant AE. Furthermore, the brain parenchyma can act as an “immunoprecipitator” of NMDAR antibodies (16), so unless the parenchyma is “saturated” by an extremely high concentration of antibody, it is plausible to expect that NSAbs may not be found in the CSF while still directly disrupting brain function.

**Figure 1:**
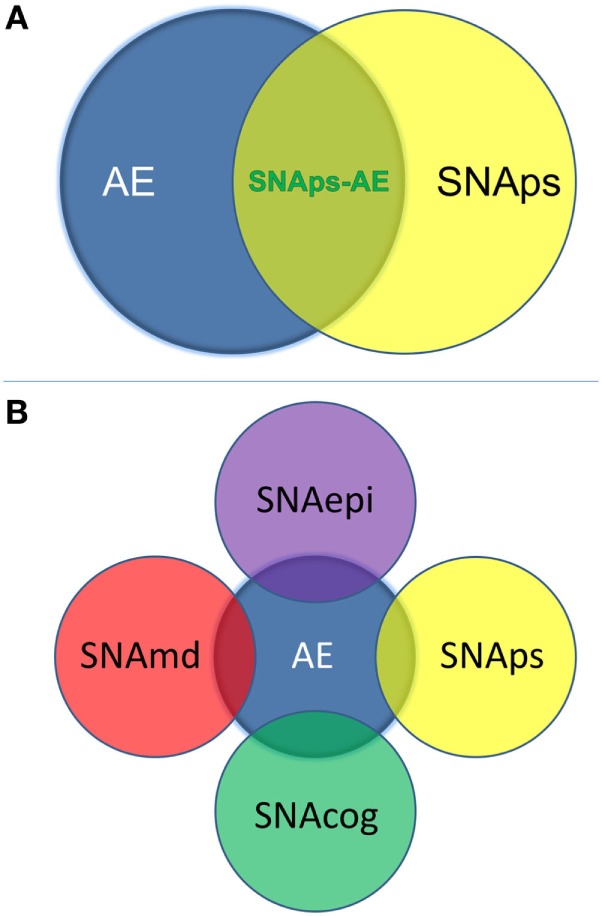
**(A)** Patients with isolated psychiatric symptoms and a detectable neural surface antibody can be characterized as having a “synaptic and neuronal autoantibody-associated psychiatric syndrome,” abbreviated to “SNAps.” This distinguishes these patients from the majority of patients with autoimmune encephalitis (AE), which is normally a multi-symptom disorder with specific associated clinical and paraclinical features: diagnostic criteria for AE have been outlined in a recent position paper (14). Some patients with isolated psychiatric symptoms will also meet criteria for AE—these patients are here referred to as SNAps-AE and are clinically atypical for AE by virtue of their monosymptomatic presentation. **(B)** The distinction between an isolated symptomatic presentation and a polysymptomatic AE presentation can usefully be extended to non-psychiatric presentations. This scheme recognizes that there will be areas of overlap where a monosymptomatic presentation meets paraclinical criteria for AE e.g. imaging, electroencephalogram, or cerebrospinal fluid parameters. Here ‘md’ stands for movement disorder, ‘cog’ is cognitive disorder, and ‘epi’ is epilepsy.

We propose that these challenges for the field can be addressed by use of a syndrome-level taxonomy. Patients with isolated psychiatric symptoms and detectable NSAb can be characterized as “synaptic and neuronal autoantibody-associated psychiatric syndromes,” abbreviated to “SNAps” (Figure 1A). SNAps deliberately have less stringent criteria for onset time, antibody class, and assay criteria. This enables a broad category, agnostic to the precise pathogenic role of the NSAb. In real time, additional serum antibody tests, identification of CSF antibody, or further investigations (e.g., MRI, EEG) may reveal abnormalities that meet the diagnostic criteria for AE, and presuming that new symptom domains do not evolve, these patients can then be characterized as having SNAps-AE (see Table 1). Patients with clear features of AE would continue to be classified as such. We suggest that this model, in which an NSAb-associated isolated clinical syndrome and AE partially overlap, can be extended to other syndromes such as epilepsy (17) and cognitive impairment (18) (see Figure 1B). Table 2 demonstrates that many of the signs and symptoms of AE are in fact seen (often to an attenuated degree) in patients with so called “isolated” psychosis, suggesting that the overlap between these conditions may be even greater still. The nascence of the field would suggest this system to be clinically pragmatic and also heuristic, promoting the delineation of biologically defined disease sub-classes and, equally as important, the signals that imply lack of disease relevance.

**Table 1:** **Comparison of position statement on diagnosis of autoimmune encephalitis (AE) and proposed SNAps concept**.

	Position paper AE diagnosis (14)	Proposed SNAps concept	

	Definite anti-NMDA receptor encephalitis	Synaptic and neuronal autoantibody-associated psychiatric syndrome (SNAps)-AE	SNAps
**Reasonable exclusion of other disorders**			
Clinical features	*Onset* <*3 months* of 1 of 6 symptom groups 1. Abnormal (psychiatric) behavior or cognitive dysfunction 2. Speech dysfunction 3. Seizures 4. Movement disorder, dyskinesias, or rigidity/abnormal postures 5. Decreased conscious level 6. Autonomic dysfunction or central hypoventilation	*Onset* <*3 months* of 1 symptom group 1. Abnormal (psychiatric) behavior	*Any onset* of 1 symptom group 1. Abnormal (psychiatric) behavior

Cerebrospinal fluid (CSF)	With or without Pleocytosis OR oligoclonal bands	With or without Pleocytosis OR oligoclonal bands	Absent

Electroencephalogram (EEG)	With or without Focal/diffuse slow or disorganized activity OR epileptic activity OR extreme delta brush	With or without Focal/diffuse slow or disorganized activity OR epileptic activity OR extreme delta brush	Absent

Magnetic Resonance Imaging (MRI)	With or without Changes suggestive of encephalitis	With or without Changes suggestive of encephalitis	Absent

Autoantibody			
• Class • Target	IgG NMDAR GluN1	IgG NMDAR GluN1	IgG OR IgM OR IgA NMDAR GluN1 NMDAR GluN1 + 2 GABA_A_R GABA_B_R AMPAR LGI1 Caspr2 OR unknown target

Sample required			
• Cell-based assay (CBA) only • CBA and confirmatory test (live neurons or immunohistochemistry)	CSF ± serum Serum	CSF ± serum Serum	Serum ± Absent

*NMDAR antibodies are the most frequently identified neural surface antibody in isolated psychiatric syndromes; therefore, NMDAR-AE offers the most useful paradigm. It is possible to make a diagnosis of NMDAR-AE if an isolated psychiatric syndrome of subacute onset is associated with CSF NMDAR (NR1) antibody or in serum if a CBA result is confirmed by testing on neuronal cultures or immunohistochemistry*.

*A case would be characterized as SNAps if there was a psychiatric syndrome of any speed of onset with serum antibody of any class against a central nervous system neuronal surface target detected on CBA. We argue that cases of NMDAR-AE with only a psychiatric syndrome share aspects of both and could be considered “SNAps-AE.” This reflects the isolated clinical syndrome atypical of AE, but paraclinical features typical of AE*.

**Table 2:** **Clinical overlap between symptoms and signs of autoimmune encephalitis (AE) and psychotic disorders**.

Clinical symptom/sign	In which AE syndrome?	Observations in psychotic disorders
Seizures	Observed in AE associated with most NSAbs	Epilepsy overrepresented in patients with schizophrenia (odds ratio 11.1) (19)

Cognitive dysfunction	Observed in AE associated with most NSAbs	Observed in schizophrenia across a range of domains. Associated with poor function and clinical outcome (20)

Movement disorders	Observed in AE associated with most NSAbs	9% of antipsychotic-naive patients with schizophrenia have spontaneous dyskinesias; 17% have spontaneous parkinsonism (21)

Catatonia	Most marked in NMDAR-AE but observed in cases of AE associated with VGKC complex antibodies and GABA_A_R antibodies	Prevalence in psychiatric patients ranges from 7.6% to 38%. 10–15% of patients with catatonia have a schizophrenia diagnosis (22)

Language disorders	Most marked in NMDAR and AMPAR AE. Catatonic speech signs such as echolalia and palilalia are also common	“Formal thought disorder” is a cardinal feature of psychotic disorders and manifests in disordered speech, sometimes called “schizaphasia”—in some cases not distinguishable from neurological dysphasia (23)

Autonomic dysfunction	Observed in AE associated with most NSAbs	Ambulatory patients with schizophrenia have mean reduced body temperature of 0.2°C (24). Meta-analytical evidence of reduced heart rate variability in psychotic disorders (25)

Hyponatremia	Observed in cases of AE associated with VGKC complex antibodies, particularly LGI1	Occurs in 6% of chronic psychiatric patients (26); polydipsia present in 3–17% of psychiatric patients (27); 40% of psychotic patients admitted with unexplained hyponatremia are not taking antipsychotic medication (28)

Antipsychotic sensitivity including rhabdomyolysis	Observed in NMDAR-AE	Neuroleptic malignant syndrome (rigidity, catatonia, confusion, hyperthermia and rhabdomyolysis) occurs in up to 0.07–2.2% of patients taking antipsychotics (29). Rhabdomyolysis can occur with water intoxication and hyponatremia

Sleep dysfunction	Observed in AE associated with most NSAbs. Particularly marked in NMDAR-AE- and IGLON-5-associated encephalopathy	Reported in 30–80% of patients with schizophrenia. Consistent findings include increased sleep onset latency, diminished slow wave sleep time, and decreased REM latency (30)

## Are NSAbs in SNAps Directly Pathogenic, Indirect Markers, or Innocent Bystanders?

Most NSAbs appear to share a common mechanism of action in their ability to cause cross-linking and internalization of their target antigen (4). Where the target is a neurotransmitter ion channel receptor, it is likely that the resulting receptor hypofunction results in impaired neurotransmitter signaling, as demonstrated by abolition of currents on some neurophysiological assays and alterations in synaptic plasticity (31, 32). Although not proven, the rationale for this causing the signs and symptoms of NSAb-associated disease is compelling and the “receptor hypofunction” model resonates well with many current theories of psychiatric disorders (33). Importantly, pathogenic potential as indexed by effects on receptor internalization and postsynaptic currents *in vitro* has been demonstrated for NMDAR antibodies taken from patients with schizophrenia (10, 34) and to a lesser extent bipolar disorder (35), demonstrating that pathogenicity is not restricted to NSAbs found in encephalitic presentations.

Synaptic dysfunction affecting glutamatergic neurotransmission has been proposed as a mechanism in schizophrenia, bipolar disorder, and major depression (36–38). We consider that antibody-mediated glutamatergic synaptic dysfunction, if relevant to psychiatric symptomatology, is likely to have relevance that cuts across traditional diagnostic boundaries. For example, the psychiatric phase of NMDAR-AE can include a diverse range of symptoms including affective and anxiety in addition to psychosis (39) and prevalence estimates detect NMDAR antibodies across traditional diagnostic boundaries (40). If NMDAR antibodies are pathogenic outside of encephalitis, then we could expect the psychiatric manifestations to have similar clinical heterogeneity. Nonetheless, only further clinicopathological correlation of SNAps will help determine an accurate picture. Such study may help validate or suggest new directions for receptor-based models of idiopathic psychiatric syndromes.

Alternatively, NSAbs in SNAps may not be directly pathogenic but still are part of the primary disease process, for example, as part of a broader immune and/or inflammatory syndrome which may be IT responsive. A randomized controlled trial could interrogate this, but to be robust would need to be designed and powered to test multiple forms of IT and detect partial responses.

Another possible role for NSAbs in SNAps is as a prognostic marker, thereby allowing disease stratification. This may include likely response to antipsychotic medication, or illness trajectory. Cohort studies would best assess this hypothesis. Suitable populations might include those at ultra-high risk or in the prodromal phase of a psychiatric disorder, in whom such biomarkers are already sorely needed.

A final potential role of NSAb is as part of a secondary process, following a separate primary disease process. The presence of NSAbs in disorders such as herpes simplex encephalitis, Alzheimer’s disease, Creutzfeldt–Jakob disease, and other dementia types (18, 41–43) strongly suggests that NSAb production sometimes occurs following neuronal destruction. Nonetheless, “secondary” antibodies can still have pathogenic potential: in dementia, NSAbs may confer a higher risk of psychosis (43), and in patients who have had herpes simplex encephalitis, NSAbs associate with greater cognitive impairment (44).

It is plausible that NSAbs associated with psychiatric disease fall into this “phenotype-modifying” category. For example, NMDAR antibodies may be found in psychosis because the primary pathology has rendered NMDARs immunogenic. Other “immunizing” conditions could include late pregnancy and parturition, around which time numerous immunological rebound changes are understood to occur (45). Indeed NSAbs have been detected in the serum of postpartum psychosis cases (13). These women responded to usual psychiatric care and clinically did not present as having AE, but even in these situations, the value of IT remains to be decided by rigorous trials.

The autoimmune encephalitides have largely been described in association with Abs of the IgG isotype, and typically only IgG is screened for clinically. While the majority of autoimmune disorders are indeed IgG-mediated, numerous instances of IgA- and IgM-mediated diseases exist outside of the CNS, such as IgA pemphigus and autoimmune hemolytic anemia. IgA and IgM NSAbs have been consistently reported in psychiatric disorders (9, 46, 47). Importantly, there is *in vitro* evidence of their pathogenicity (18, 34, 35), and IgA and IgM seropositivity does appear to associate with clinical phenotype in some non-encephalitis conditions (18). We suggest that it is premature to dismiss *a priori* non-IgG NSAbs as irrelevant to the disease. For the purposes of our categorical model, IgA or IgM seropositivity is included in the definition of SNAps, but not of SNAps-AE, implying possible, but not definite, causal relevance.

Animal models could potentially demonstrate the pathogenicity of NSAbs in SNAps, but animal models of AE have been slow to develop and often recapitulate a limited facet of a complex phenotype (48, 49). The abnormal movements, spontaneous seizures, autonomic instability, or psychosis-like behaviors associated with AE are notably absent. Nonetheless, the animal model of NMDAR encephalitis of Planaguma and colleagues (48) appears to be an unintended but plausible model of NMDAR antibody-mediated depressive behavior. Future work on an animal model of SNAps should attempt to integrate passive NSAb transfer experiments with established psychiatric animal endophenotypes. For example, in a potential model of psychosis, multiple behavioral and neurophysiological indices such as latent inhibition and mismatch negativity could be assessed alongside the neuronal effects of antibodies (50).

Ultimately, *in vivo* human studies of SNAps will be necessary to elucidate whether NSAbs differentially impact brain function in psychiatric presentations. Functional MRI and magnetic resonance spectroscopy offer equivalent promise in restricted psychiatric presentations as for more fulminant neurological presentations (51, 52). With the development of PET and SPECT ligands for *in vivo* measurement of microglial activation (53, 54) and the function of individual receptor types [e.g., NMDAR (55) and GABAR (56)] or neurotransmitter synthesis capacity (57), it is likely that this methodology can offer insights into the molecular pathology of SNAps. Correlation of clinical improvement with improvement in disease-relevant biomarkers (for example, a perturbed glutamate MRS signal or ketamine-like functional dysconnectivity) following antibody removal would strengthen an argument for *in vivo* pathogenicity in SNAps. Conversely, an absence of differences from seronegative patients with psychiatric disorders on such neuroimaging measures would make it less likely that NSAbs directly affect brain function.

### Does the Blood–Brain Barrier (BBB) Have a Role?

As a number of studies have reported similar seroprevalences in NSAbs in individuals with multiple psychiatric diseases and healthy controls, some authors have postulated that disruption of the BBB must be present for NSAbs to be pathogenic (9).

Beyond defining what “BBB disruption” actually means (disruption of tight junctions, permeability to macromolecules, and hyper- or hypofunction of transporter mechanisms have all been suggested), it is difficult to demonstrate *in vivo*. Serum markers, such as calcium-binding glial protein S100B, appear to correlate well with BBB disruption in some conditions but not others (58). Until recently, neuroimaging approaches were only able to reveal gross BBB disruption, but newer dynamic contrast-based techniques may allow for the identification of more subtle impairments (59). The need for a simultaneous lumbar puncture and blood test makes the gold standard test, CSF/serum albumin ratio (Qalb), difficult to obtain in psychiatric practice.

Proxy markers of BBB disruption may have to suffice. Work by Ehrenreich and colleagues have demonstrated that only in the presence of a history of birth complications and “neurotrauma” does NMDAR seropositivity in schizophrenia predispose to more severe neurological symptoms (10). Additionally, drawing on animal work demonstrating that ApoE4 carriers have a chronically “leaky” BBB, Hammer and colleagues demonstrated that patients with schizoaffective disorder show a higher than expected co-occurrence of NMDAR Abs and ApoE4 carrier status compared to patients with other psychiatric diagnoses and healthy controls, suggesting that in seropositive individuals, a leaky BBB confers susceptibility toward a schizoaffective phenotype (60).

**Figure 2:**
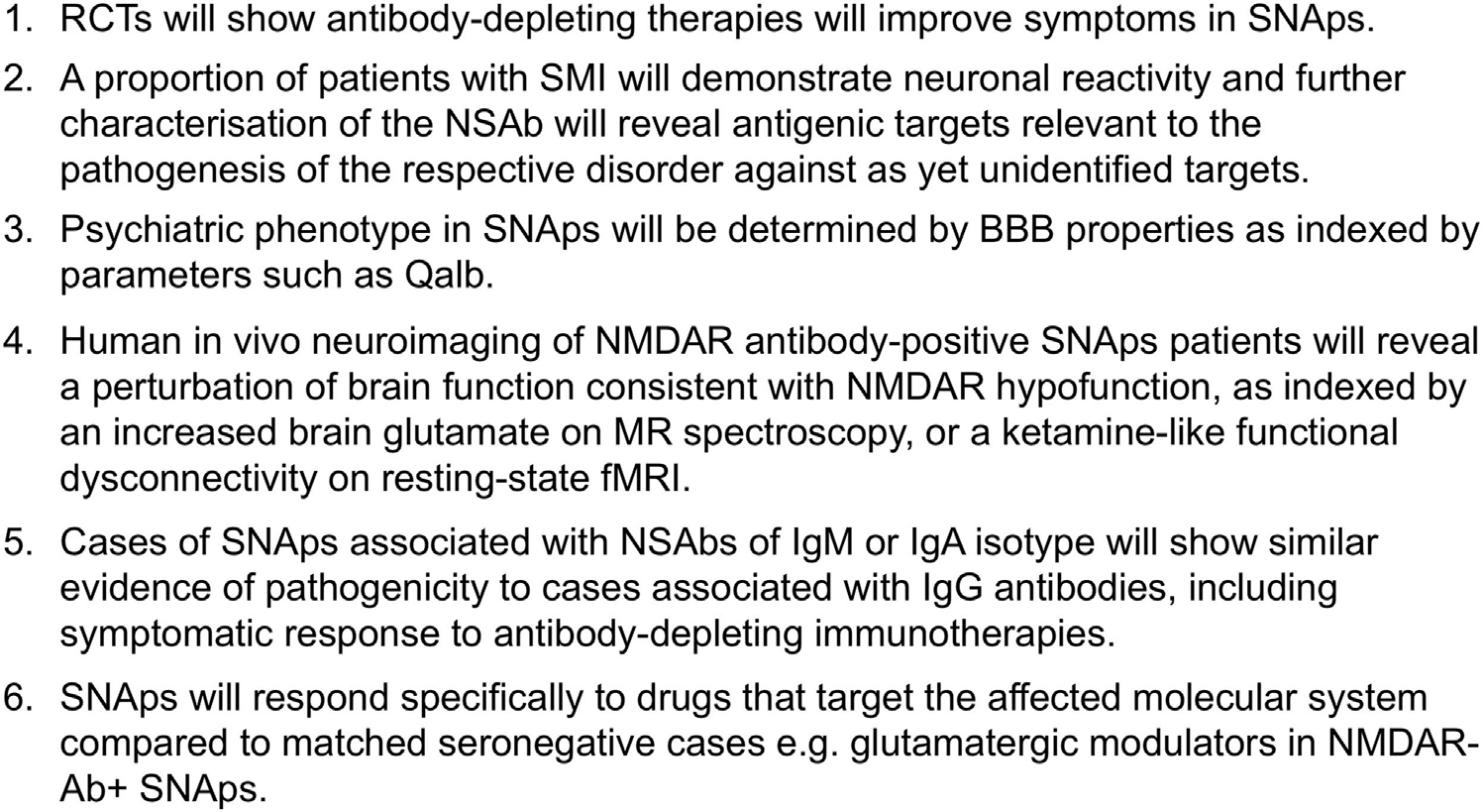
**Experimentally testable hypotheses relating to the pathogenicity of neural surface antibodies (NSAbs) in synaptic and neuronal autoantibody-associated psychiatric syndromes (SNAps)**.

We suggest that Qalb or dynamic contrast-enhanced imaging is used in prospective studies of SNAps where possible, to explore the potentially disease-mediating role of the BBB (see Figure 2).

## How Can We Integrate These Findings into Psychiatric Practice?

### Who Should Be Tested for NSAbs, and Which Tests Should Be Requested?

The need to identify cases of possible AE at an early stage in psychiatric practice is not controversial. However, the breadth of screening is a matter of active discussion. Many cases of definite AE will, even early in their course, have clinical features that prompt presentation to medical services where the likelihood of neurological evaluation including NSAb testing is higher. However, in diseases such as NMDAR-AE, psychiatric symptoms may predominate for the first weeks (or sometimes months) of the illness (61) and occasionally may not progress any further (62) (this is a situation that we have referred to as SNAps-AE in Figure 1 and Table 1).

It is likely that many of these cases satisfy current criteria for “possible AE” as per Graus et al. (14), and some that eventually go on to receive a diagnosis of “probable” or “definite” NMDAR-AE are initially diagnosed as having a primary psychiatric disorder and are at risk of suboptimal management.

**Figure 3:**
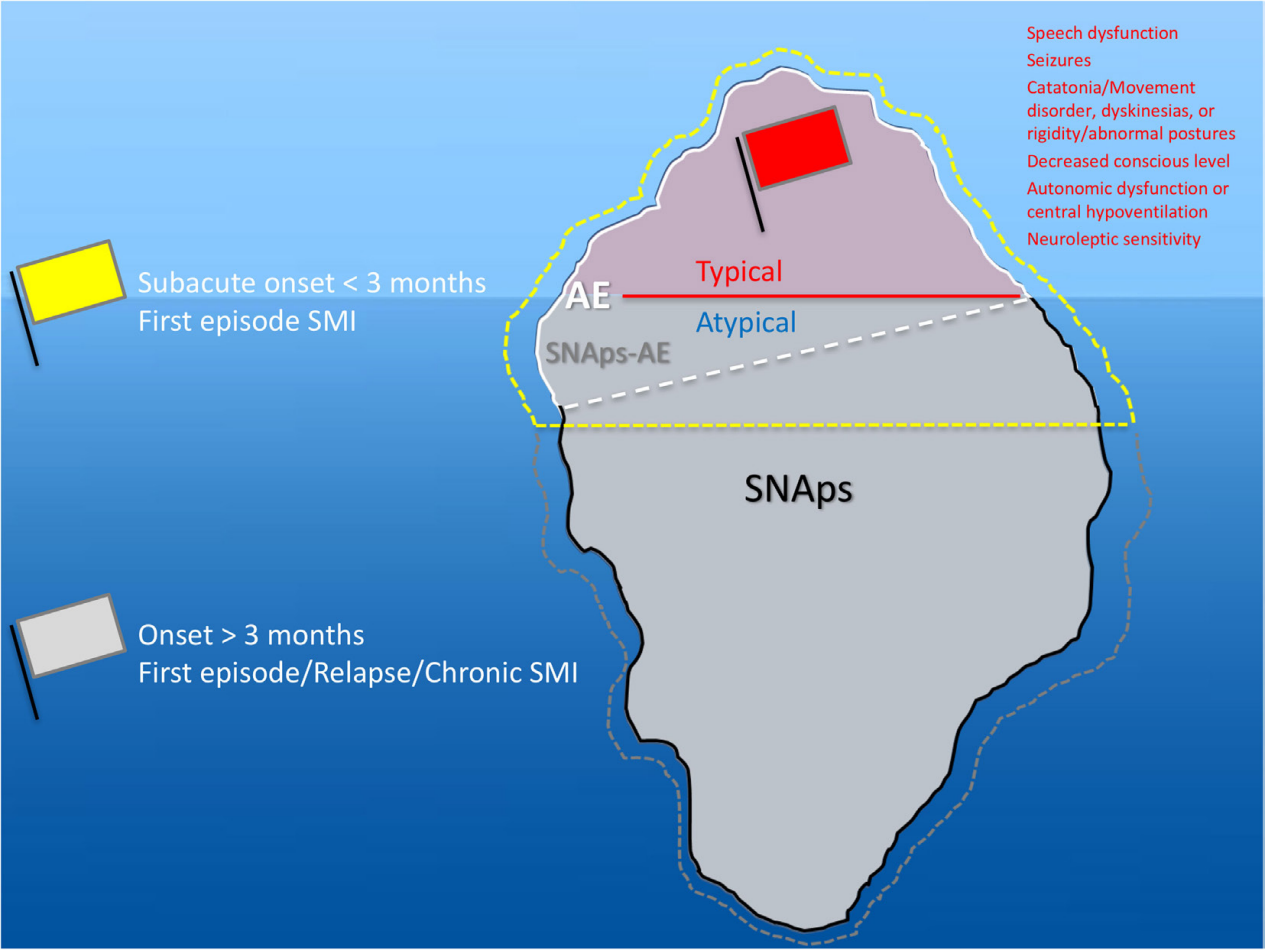
**Relationship between severe mental illness (SMI) and the neural surface antibody (NSAb) seropositivity iceberg: psychiatrists will see both autoimmune encephalitis (AE), and synaptic and neuronal autoantibody-associated psychiatric syndromes (SNAps), and overlap areas**. In practice, this means that all first-episode SMI with a subacute onset should be regarded as a yellow flag for AE and should be screened for relevant NSAbs (see Figure 4). If these cases have red flag clinical features, then there should be a low threshold for further investigations and liaison with neurology colleagues (see Figure 5). Screening cases of SMI with a longer onset or treatment resistance will yield cases with NSAbs; however, the management of these cases is less certain. There is an imperative for further well-designed research studies to characterize the biology and immunotherapy responsiveness of these cases.

**Figure 4:**
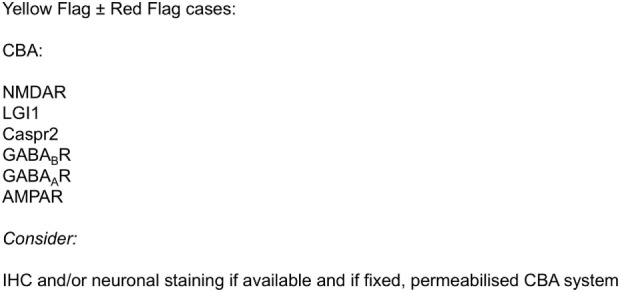
**Initial serum neural surface antibody panel recommended in subacute onset first-episode severe mental illness, at risk for autoimmune encephalitis, and to consider in cases with longer onset**.

Although prior to the discovery of AE it is likely that some cases spontaneously remitted (63–65), there is clear, albeit necessarily observational, evidence that short- and long-term medical and neuropsychiatric prognosis is linked to early clinical identification of AE and timely instigation of IT, frequently before the antibody status is known (66). Therefore, we suggest that cases of FEP or severe mood disturbance such as mania or severe depression, here grouped as “severe mental illness” (SMI), with subacute onset (less than 3 months), should be regarded as “yellow flag” cases, at risk of AE, and undergo testing for a serum NSAb panel. Cases with both yellow and additional “red flag” clinical features suggestive of AE should be more obvious to differentiate clinically, and likelihood of detection of relevant NSAb detection is high (see Figures 3 and **4**).

**Figure 5:**
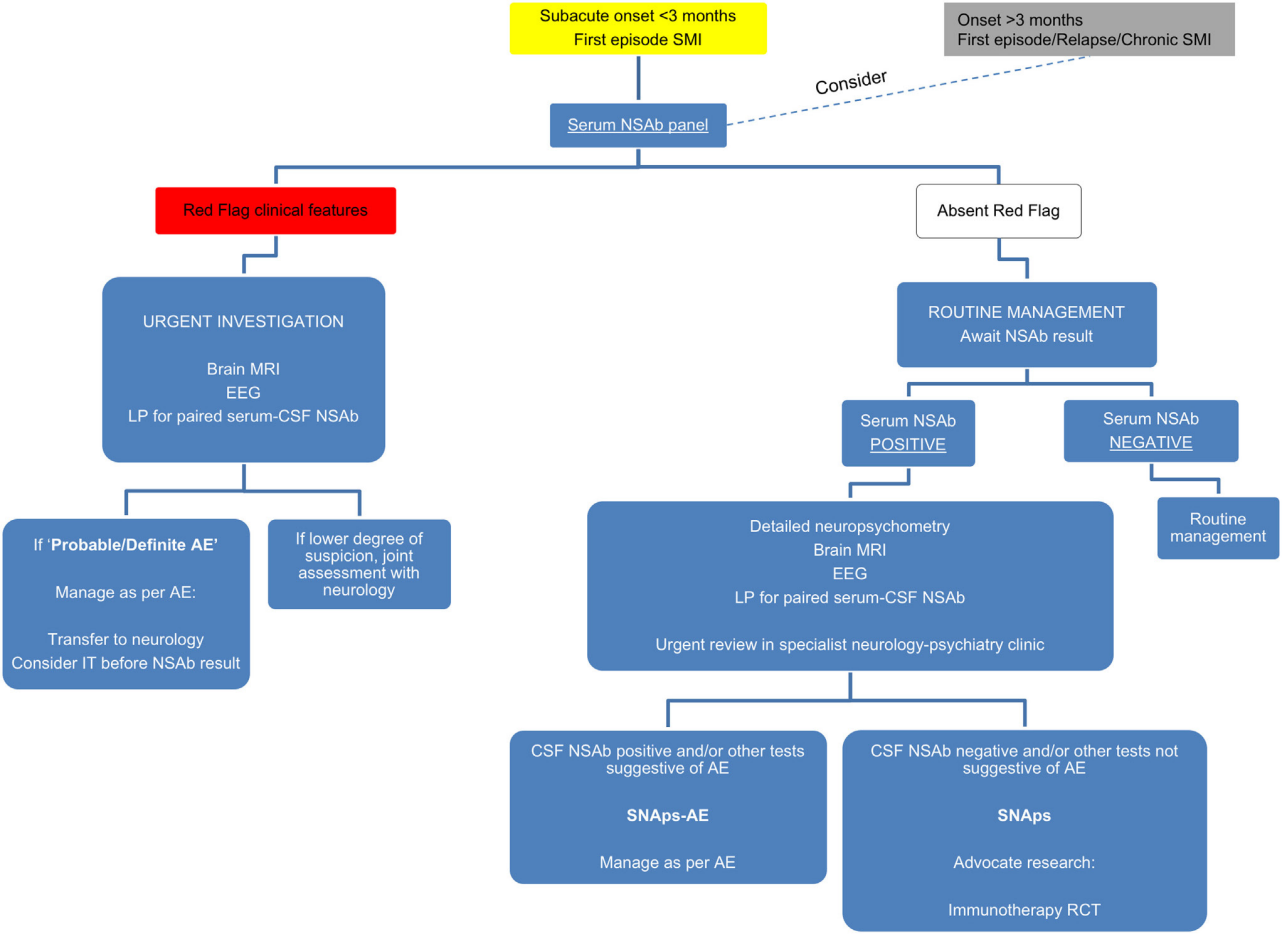
**Suggested algorithm for neural surface antibody (NSAb) testing and further management in the context of severe mental illness (SMI)**.

For cases of SMI with (a) an onset of longer than 3 months, (b) in relapse, or (c) in a chronic phase—referred to as “gray flag” cases—there is growing evidence to suggest that many of these cases will have NSAbs without differentiating clinical features (67), and that many of these may stand to benefit from IT (68). However, blanket screening of such cases outside of clearly defined research programs risks generating uncertainty insofar as antibody-positive patients may be identified before a clear and evidence-based understanding of their optimal management is known. Therefore, we would strongly advocate screening in these cases to be based within research settings (see Figure 5).

### How Should Psychiatrists Understand a Positive Test?

Local experience has found that NSAb screening in subacute onset first-episode presentations and more long-standing instances of serious mental illness (SMI) helps to identify cases of AE that will benefit from IT. However, as with any screening test, there will be positive results of less clear clinical significance. For example, in those in whom illness does not clearly satisfy criteria for AE, or is spontaneously remitting, or is responding well to psychological or psychotropic approaches.

We would advocate that, where possible, cases of SNAps are assessed in detail in a specialist joint neurology–psychiatry clinic. Such services will often be based at research-associated regional neuroscience centers with access to neuropsychology, brain imaging (MRI and increasingly PET), and lumbar puncture/CSF analysis expertise.

We suggest taking a pragmatic approach incorporating available resources and tolerability to investigation. We hope this could prompt clinicians to consider how psychiatric services could incorporate the developments from this rapidly growing field. Specialist clinical work closely allied with multidisciplinary research units will be central in identifying biomarkers of future classificatory and clinical relevance (summarized in Figure 5).

### Who Should Receive NSAb-Modifying IT?

While the evidence for short- and long-term benefits for IT in AE is clear, clinicians managing SNAps have greater equipoise. If NSAbs in these cases are indeed pathogenic, care must be taken to minimize the duration of untreated autoimmune CNS disease. However, the medical risks of IT are not trivial, and pathogenicity of NSAbs in SNAps has not been demonstrated definitively. Although open-label data (68) and many case reports (7, 69–71) show promising effects of IT in cases of psychosis, these results do not exclude placebo response or regression to the mean. Duration of adequate treatment and optimal treatment of relapses is not clear.

In our experience, there is another potential hazard that can arise when discussing the possible relevance of NSAbs with seropositive patients: in some cases, the patient and/or their carer may develop unhelpful biomedically reductionist illness beliefs (e.g., “I don’t have a mental health problem. I have encephalitis. That’s what the antibody test shows.”) which prevent engagement in vital psychosocial interventions.

Given such equipoise, there is a clear and pressing need for adequately powered and robustly designed randomized controlled trials to determine whether SNAps cases benefit from IT.

### What Other Treatments Should Be Considered?

Identifying SNAps may also allow better targeting of existing treatments. For example, sensitivity to antipsychotic medication and propensity to neuroleptic malignant syndrome-type complications occur relatively commonly in AE and overlap areas (72, 73). In some cases of AE, patients experience treatment-resistant psychiatric symptoms despite IT, and several reports note the efficacy of electroconvulsive therapy (ECT) in this situation (74). The mechanism of this, in the context of an identified molecular pathology, is intriguing. From a clinical perspective, it implies that ECT may be a specific intervention worth considering early in SNAps.

Also intriguing is early evidence of SNAps acting as a paradigm for intervention with rational molecular-based psychopharmacology. For example, Heresco-Levy and colleagues found that the NMDAR co-agonist d-serine could improve psychopathology in a case of chronic treatment-resistant schizophrenia with NMDAR antibodies (75). This approach could be widened to other antibodies implicated in SNAps.

## Conclusion

The description of AE syndromes caused by NSAbs has profoundly impacted neurological practice and has invigorated neuroimmunology as a basic and clinical science. The extent to which the paradigm—of autoantibodies affecting brain function and behavior—has relevance for clinical psychiatry is a matter of considerable debate.

We have introduced the concept of SNAps as an attempt to clarify some of the issues in this sometimes confusing field. By making the distinction between AE and isolated psychiatric presentations associated with NSAbs, we hope to encourage the latter as an important focus of research in its own right. It is now generally accepted that many psychiatric disorders may be comprised of subgroups that are phenotypically similar but heterogeneous in their etiologies. We believe that the category of SNAps offers a well-defined candidate for one such subgroup, and we have offered suggestions as to how this hypothesis may be tested.

Research on SNAps will of course be informed by the AE literature to date, particularly in terms of pathogenic mechanisms. In this article, we have outlined the increasingly compelling evidence that NSAbs may have pathogenic potential in SNAps: we are clear however that considerable further work needs to be done in this area before generalized statements can be made about pathogenicity. Further, we would suggest that much of this work needs to be led by psychiatrists. The nuances of psychiatric signs and symptoms are often ignored within the neurological literature, and while this remains the case, a “psychiatric phenotype” associated with NSAbs will remain elusive.

The possibility that the SNAps concept may delineate a subgroup of psychiatric patients with a differential treatment response (including a potential IT response) remains an exciting focus of future research. In an area of medicine where novel therapies are relatively rare and where immune therapies are increasingly under the spotlight, SNAps represent a focus for therapeutic studies that could be potentially transformative for the field.

## Author Contributions

AA-D, TP, AL, and BL conceived the article. AA-D, TP, and AL drafted the text. AA-D and TP prepared the tables and figures. BL revised the text, tables, and figures. All the authors read and approved the final manuscript.

## Conflict of Interest Statement

The authors declare that the research was conducted in the absence of any commercial or financial relationships that could be construed as a potential conflict of interest.
